# Smoking and quitting behaviours by mental health conditions in Great Britain (1993–2014)

**DOI:** 10.1016/j.addbeh.2018.10.011

**Published:** 2019-03

**Authors:** Sol Richardson, Ann McNeill, Leonie S. Brose

**Affiliations:** a King's College London, Addictions Department, Institute of Psychiatry, Psychology & Neuroscience, 4 Windsor Walk, London SE5 8BB, United Kingdom; b UK Centre for Tobacco and Alcohol Studies, United Kingdom

## Abstract

Smoking is a major contributor to the disparity in life expectancy between those with and without a mental health condition. Previous work has found associations between individual conditions such as depression and current smoking, cigarette consumption and dependence, but did not compare a range of specific mental disorders. Using data from the nationally-representative Adult Psychiatric Morbidity Survey, we characterised trends in smoking prevalence in the general population in Great Britain and among those with and without mental health conditions for the period 1993–2014. We tested associations across different common mental health conditions (including depression, phobia, generalised anxiety and mixed anxiety and depression), in addition to personality conditions, and heaviness of smoking, desire to quit, perceived difficulty of remaining abstinent and successful cessation within the previous 12 months. Smoking prevalence among those without any mental health condition decreased from 29.3% in 1993 to 19.6% in 2014. Prevalence was higher among those with a condition but fell from 44.6% to 34.1%. Having a mental health condition was associated with current smoking, heavy smoking, difficulty remaining abstinent, desire to quit and perceived difficulty remaining abstinent. The same was found for all conditions individually but the strength and significance of the associations varied. Having any common mental health condition was associated with lower odds of smoking cessation—but not after adjustment for heavy smoking. We found no significant associations between individual conditions and cessation outcomes, however. In summary, smoking prevalence among people with common mental health conditions remained around 50% higher than among those without despite their higher desire to quit. Adequately addressing higher dependence could support cessation and contribute to narrowing health disparities.

## Highlights

• First population-level study of smoking, quitting and mental health within the UK
• From 1993 to 2014, smoking prevalence fell in those with and without a condition.
• Prevalence remained >50% higher among those with a mental health condition.
• Those with conditions were more likely to smoke but had higher desire to quit.
• Their lower odds of cessation diminished after adjustment for heavy smoking.

## 1 Introduction

There exist significant inequalities in health and life expectancy between individuals with mental health conditions and the general population (Chang et al., 2011; Chesney, Goodwin, & Fazel, 2014; Thornicroft, 2013) which have been estimated at up to 12.0 years for women and 15.9 years for men (Lawrence, Hancock, & Kisley, 2013). The difference in smoking prevalence between these groups is a major contributor to these persistent inequalities which can account for up to two thirds of the disparity in life expectancy (Tam, Warner, & Meza, 2016).

Previous evidence has shown that individuals with various types of mental health conditions are more likely to be smokers, more likely to be heavy smokers, and more likely to be highly dependent on cigarettes (Anda et al., 1990; Bowden, Miller, & Hiller, 2011; Brown, Madden, Palenchar, & Cooper-Patrick, 2000; de Leon, Becoña, Gurpegui, Gonzalez-Pinto, & Diaz, 2002; de Leon & Diaz, 2005; Glasheen, Hedden, Forman-Hoffman, & Colpe, 2014; Hagman, Delnevo, Hrywna, & Williams, 2008; Lasser et al., 2000; Lawrence, Mitrou, & Zubrick, 2009; Lipari & Van Horn, 2017; Matcham et al., 2017; Morris, Giese, Turnbull, Dickinson, & Johnson-Nagel, 2006; Royal College of Physicians, Royal College of Psychiatrists, 2013; Szatkowski & McNeill, 2015). Studies have found either no association (Prochaska et al., 2011; Matcham et al., 2017) or even a positive association (Anzai, Young-Wolff, & Prochaska, 2015) between mental health conditions and desire to quit among smokers. Negative associations have also been found between having mental health conditions and odds of successful smoking cessation (Glassman et al., 2001; Anda et al., 1990; Lasser et al., 2000; de Leon & Diaz, 2005; Hagman et al., 2008; McClave, McKnight-Eily, Davis, & Dube, 2010; Glasheen et al., 2014). Although together these studies investigated various conditions, including depression, schizophrenia and anxiety, and their relationship with smoking-related outcomes, most only considered a single condition. The lack of comparable evidence on the associations between different specific conditions and their associations with smoking-related behaviours represents an important research gap and a missed opportunity to inform whether individuals with different conditions may face different barriers to successful cessation. Specifically, although there are differences in smoking prevalence between individuals with different conditions (Royal College of Physicians, Royal College of Psychiatrists, 2013), to our knowledge no previous work has compared differences in other smoking-related behaviours such as cessation.

The objectives of this paper were to characterise the population-level trends in smoking prevalence among adults with and without a common mental health condition in Great Britain using a nationally-representative data sample, and analyse associations between specific conditions and outcomes including smoking status, heavy smoking and quitting behaviours at the individual level in 2000 to elucidate the explanatory factors underlying the population-level trends.

## 2 Materials and methods

Data were obtained from the Adult Psychiatric Morbidity Survey (APMS), a representative survey of psychiatric morbidity among adults in private households in Great Britain administered by the Office for National Statistics (ONS) on behalf of the Department of Health, the Scottish Executive and the National Assembly for Wales (Meltzer, Gill, Petticrew, & Hinds, 1995). Four waves are currently available (1993, 2000, 2007 and 2014) and sample sizes per wave range from 7403 to 10,108. Smokers were defined as respondents who replied “yes” to the question “do you smoke cigarettes at all nowadays?” Ex-smokers were defined as those who had reported ever trying a cigarette and were not currently smoking as per the pre-2016 ONS definition^1^ (ONS, 2017). The 2000 wave of APMS included variables pertaining to respondents' smoking behaviour including quantity smoked and cessation.

Respondents were categorised as having a mental health condition if they met the criteria for any common mental health condition (MHC) on the revised Clinical Interview Schedule (CIS-R) within the last week. These included depression, phobia, generalised anxiety disorder (GAD), panic disorder, obsessive-compulsive disorder (OCD) and mixed anxiety and depressive disorder. Mixed anxiety and depression is a ‘catch all’ category encompassing individuals with a score of ≥12 on the CIS-R who did not meet criteria for any of the other categories for neurotic conditions (Lewis, Pelosi, Araya, & Dunn, 1992). Panic disorder and OCD were not tested separately as they comprised <3% of the sample with a MHC. The analyses used data from respondents aged 16–64 years as individuals outside this range were excluded from the 1993 wave. All analyses were performed in Stata 15 and applied cross-sectional survey weights.

We estimated weighted proportions of smokers, ex-smokers and never-smokers among the population of Great Britain in each available wave of APMS, and then among those with and without a MHC. The proportion of smokers in each wave with a MHC was also estimated.

We performed logistic regression analyses to test the associations between a respondent meeting the criteria for any MHC, depression, phobia, GAD, OCD or mixed anxiety and depression compared with those who did not, and six different outcomes using data from the 2000 wave of APMS only.^2^ Outcomes included (i) current smoking status, (ii) heavy smoking (≥20 cigarettes/weekday), (iii) dependence defined as a moderate or high Heaviness of Smoking Index (HSI) score (≥3) (Borland, Yong, O'Connor, Hyland, & Thompson, 2010; Heatherton, Kozlowski, Frecker, Rickert, & Robinson, 1989), (iv) desire to quit^3^, (v) perceived difficulty of remaining abstinent for one day^4^, and (vi) successful smoking cessation within the previous 12-month period^5^.

Models were fitted for each CIS-R diagnostic category and outcome. Model 1 tested the unadjusted associations between CIS-R categories and each outcome. Models were then adjusted for socio-demographic variables including gender, current age, ethnicity, partnership status, housing tenure, occupational position, level of education and age when the respondent first started smoking (if applicable) (Model 2). Models for desire to quit, difficulty remaining abstinent and 12-month smoking cessation were then further adjusted for heavy smoking^6^ (<20 or ≥20 cigarettes/weekday) currently or before successful cessation as appropriate (Model 3). The same procedures were used to test associations between personality conditions and smoking-related outcomes (see Supplementary Note).

## 3 Results

**Graph 1:**
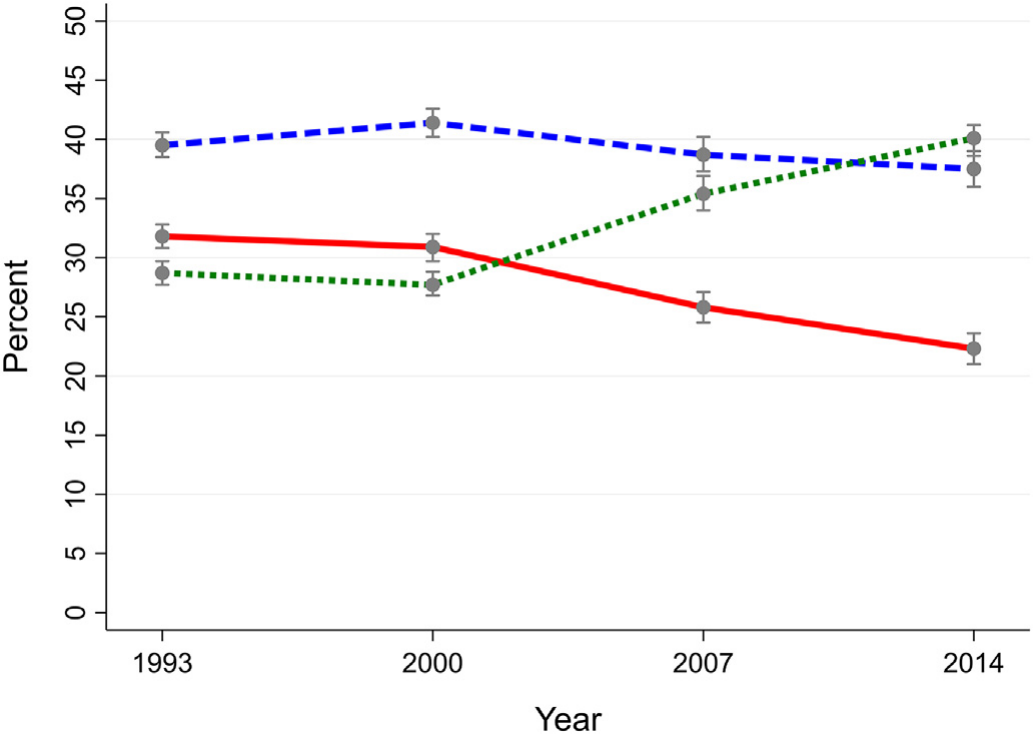
Overall British population. Proportions of smokers (solid line), ex-smokers (dashed line) and never-smokers (dotted line) in the British population aged 16–64 years overall and according to presence of mental health conditions (1993–2014).

**Graph 2:**
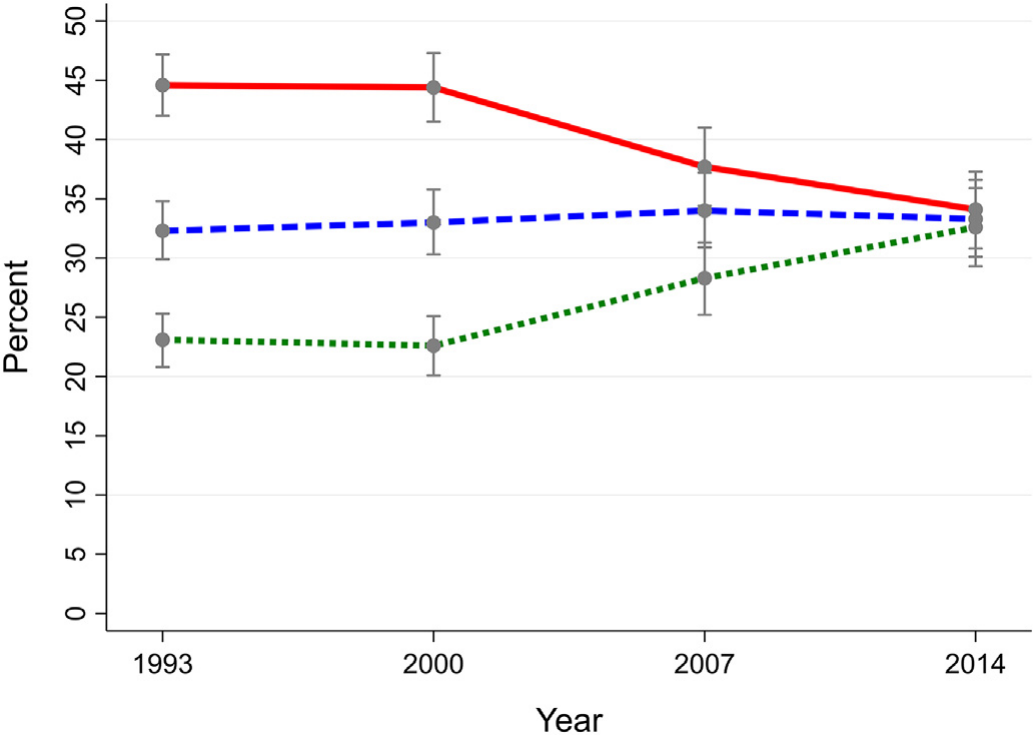
Individuals with a CIS-R common mental health condition. Proportions of smokers (solid line), ex-smokers (dashed line) and never-smokers (dotted line) in the British population aged 16–64 years overall and according to presence of mental health conditions (1993–2014).

**Graph 3:**
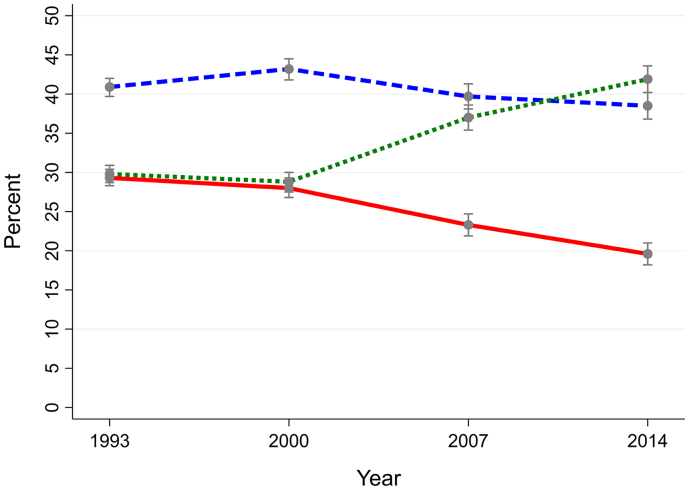
Individuals without a CIS-R common mental health condition. Proportions of smokers (solid line), ex-smokers (dashed line) and never-smokers (dotted line) in the British population aged 16–64 years overall and according to presence of mental health conditions (1993–2014).

Smoking prevalence in the general population in Great Britain decreased from 31.8% in 1993 to 22.3% in 2014, and from 29.3% to 19.6% for those without a MHC, as evidenced by the non-overlapping 95% CIs. Prevalence remained over 50% higher among those with a MHC, but fell from 44.6% to 34.1% in the same period. The proportion of never-smokers in the general population exceeded that of current smokers by 2007 and ex-smokers by 2014. The proportion of smokers who had a MHC increased over the period studied from 22.3% (95% CI: 20.9–23.9) in 1993 to 24.3% (95% CI: 22.5–26.1) in 2000, 24.5% (95% CI: 22.2–26.8) in 2007, and 28.8% (95% CI: 25.9–31.7) in 2014 (See Graph 1, Graph 2, Graph 3). Although the proportion of ex-smokers in the general population remained constant or even declined over the study period, the proportion of ex-smokers as a proportion of ever-smokers increased over time (increasing from 41.7% to 49.4% among those with a MHC and from 57.1% to 66.2% among those without a MHC from 1993 to 2014).

Of the 7306 respondents in the 2000 wave with data on smoking status and MHCs, 2360 (32.3%) were current smokers and 1371 (18.8%) had any MHC^7^. The adjusted odds of current smoking (Model 2 OR: 1.79, 95% CI: 1.56–2.07, *p* < .001), heavy smoking, moderate or high dependence and difficulty remaining abstinent were significantly higher among respondents with any MHC than those without (Table 1). Those with any MHC were more likely to report a desire to quit smoking even after adjustment for heavy smoking. Although odds of successful smoking cessation were significantly lower in this group (Model 2), the association was no longer significant after adjustment for heavy smoking (Model 3 OR: 0.82, 95% CI: 0.55–1.22, *p* = .317).

**Table 1:** Odds of current smoking, heavy smoking, dependence (moderate or high HSI score), desire to quit and successful smoking cessation by presence of common mental health conditions among individuals aged 16–64 years in the Adult Psychiatric Morbidity Survey (2000).

Model	Any MHC^1^		Depressive episode		Phobia		Generalised anxiety disorder		Mixed anxiety/depression	
	OR (95% CI)	p	OR (95% CI)	p	OR (95% CI)	p	OR (95% CI)	p	OR (95% CI)	p
i. Current smoking (all respondents, *n* = 7003)										
Model 1 (unadjusted)	2.04 (1.78–2.33)	<0.001	2.69 (2.01–3.61)	<0.001	2.52 (1.77–3.60)	<0.001	1.92 (1.53–2.40)	<0.001	1.69 (1.41–2.02)	<0.001
Model 2^2^	1.79 (1.56–2.07)	<0.001	2.03 (1.50–2.76)	<0.001	1.76 (1.20–2.59)	0.004	1.65 (1.30–2.10)	<0.001	1.63 (1.36–1.97)	<0.001
ii. Heavy smoking (current smokers, *n* = 2253)										
Model 1 (unadjusted)	1.48 (1.19–1.83)	<0.001	1.48 (0.97–2.25)	0.067	1.73 (1.05–2.84)	0.031	1.41 (1.00–1.98)	0.052	1.36 (1.02–1.82)	0.035
Model 2^2^	1.38 (1.09–1.75)	0.008	1.30 (0.85–1.97)	0.226	1.29 (0.76–2.19)	0.341	1.12 (0.78–1.61)	0.537	1.37 (1.00–1.87)	0.050
iii. Dependence (moderate or high HSI score) (current smokers, *n* = 2249)										
Model 1 (unadjusted)	2.05 (1.66–2.53)	<0.001	2.09 (1.39–3.15)	<0.001	2.08 (1.26–3.42)	0.004	2.78 (1.97–3.90)	<0.001	1.55 (1.18–2.05)	0.002
Model 2^2^	1.86 (1.48–2.33)	<0.001	1.76 (1.16–2.67)	0.008	1.48 (0.87–2.53)	0.152	2.23 (1.54–3.24)	<0.001	1.50 (1.12–2.01)	0.007
iv. Desire to quit (current smokers, *n* = 2240)										
Model 1 (unadjusted)	1.44 (1.13–1.83)	0.003	1.09 (0.69–1.72)	0.710	1.15 (0.68–1.95)	0.597	1.57 (1.03–2.39)	0.035	1.39 (1.01–1.92)	0.043
Model 2^2^	1.43 (1.12–1.83)	0.004	1.08 (0.69–1.71)	0.736	1.13 (0.67–1.92)	0.642	1.56 (1.03–2.39)	0.038	1.36 (0.99–1.89)	0.062
Model 3^3^	1.43 (1.11–1.83)	0.005	1.08 (0.68–1.70)	0.746	1.13 (0.67–1.92)	0.651	1.56 (1.02–2.38)	0.039	1.36 (0.98–1.89)	0.064
v. Difficulty remaining abstinent (*n* = 2242)										
Model 1 (unadjusted)	1.88 (1.51–2.34)	<0.001	2.58 (1.60–4.17)	<0.001	2.03 (1.19–3.47)	0.009	2.36 (1.62–3.45)	<0.001	1.38 (1.04–1.83)	0.025
Model 2^2^	1.74 (1.38–2.20)	<0.001	2.47 (1.53–4.01)	<0.001	1.67 (0.95–2.94)	0.075	2.05 (1.37–3.06)	<0.001	1.30 (0.96–1.77)	0.090
Model 3	1.65 (1.30–2.10)	<0.001	2.55 (1.56–4.15)	<0.001	1.60 (0.86–2.98)	0.138	2.01 (1.39–3.17)	<0.001	1.21 (0.88–1.66)	0.245
vi. 12-month quit ratio (ever-smokers in last 12 months, *n* = 3789)										
Model 1 (unadjusted)	0.66 (0.46–0.95)	0.027	0.71 (0.33–1.55)	0.392	1.28 (0.56–2.88)	0.558	0.57 (0.29–1.15)	0.119	0.71 (0.43–1.17)	0.182
Model 2^2^	0.64 (0.43–0.94)	0.022	0.77 (0.36–1.67)	0.512	1.34 (0.61–2.94)	0.468	0.58 (0.29–1.18)	0.133	0.66 (0.39–1.11)	0.119
Model 3	0.82 (0.55–1.22)	0.317	1.14 (0.51–2.53)	0.746	1.61 (0.72–3.59)	0.242	0.71 (0.35–1.45)	0.350	0.81 (0.47–1.40)	0.451

1 Including depressive episode phobia, generalised anxiety disorder, obsessive compulsive disorder, panic disorder and mixed anxiety and depressive disorder.

2 Adjusted for gender (binary: male or female), current age (categorical: <25, 25–34, 35–44, 45–54 or 55–64), ethnicity (binary: white or other), partnership status (categorical: married, single, separated, divorced or widowed), housing tenure (categorical: outright ownership, ownership with outstanding mortgage, or rented/other), occupational position (binary: Registrar-General's classification manual or non-manual occupation), level of education (categorical: degree-level, higher vocational (i.e. HND, teaching), A-level, GCSE or equivalent, or no qualifications) and age when first started smoking (categorical: never smoked regularly, <10, 10–14, 15–19, 20–24 or >25). ^3^Adjusted for all covariates included in Model 2 in addition to heavy smoking (binary: <20 or ≥20 cigarettes/weekday).

We also found statistically significant relationships between each of the four individual conditions tested and current smoking and moderate or high dependence; although the association of the latter with phobia was attenuated in the adjusted model. Only mixed anxiety and depression was found to have a significant relationship with heavy smoking after adjustment. GAD was significantly associated with both desire to quit and perceived difficulty remaining abstinent in the adjusted model while depression was also associated with the latter. No individual condition was significantly associated with 12-month cessation outcomes. Results for the associations between personality conditions and smoking-related outcomes are shown in the Supplementary Note.

## 4 Discussion

We found a consistent and significant decline in smoking prevalence from 1993 to 2014 regardless of presence of MHCs. Promisingly, smoking prevalence decreased by around ten percentage points among those both with and without MHCs. Smoking prevalence among those with a MHC remained notably higher, however, with little indication of convergence. Estimates of smoking prevalence among people with mental health conditions using APMS 2014 data were comparable with those from other sources (Royal College of Physicians, Royal College of Psychiatrists, 2013). For example, Health Survey for England data estimated a smoking prevalence of 37.4% (95% CI: 31.9–43.1) among people reporting a longstanding mental health condition in 2010. People with MHCs in Great Britain had higher odds of smoking, heavy smoking, dependence, having desire to quit and perceiving abstinence as difficult while having lower odds of successful cessation (but not after adjusting for heavy smoking). Certain personality conditions were associated with higher odds of smoking, cigarette dependence and perceived difficultly remaining abstinent, but also greater desire to quit.

Smoking prevalence among the population aged 16–64 is a function of individuals entering the smoking population on the one hand through initiation, relapse and younger smokers turning 16, and exiting on the other through smoking cessation, death and smokers ageing and falling out of the included age range (West & Brown, 2015). This suggests that while increasing rates of smoking cessation among those both with and without a MHC contributed somewhat to the decline in smoking prevalence in the population aged 16–64, the most important reason for the overall decline was decreasing smoking initiation and replacement of older smokers as evidenced by the rising proportion of never-smokers (Hiscock, Bauld, Amos, & Platt, 2012). The higher smoking prevalence among those with a MHC is influenced both by lower rates of smoking cessation and higher rates of initiation given the lower proportion of both never-smokers and ex-smokers in this group.

Encouragingly, and in agreement with previous findings (Siru, Hulse, & Tait, 2009), we found that smokers with MHCs (and certain personality conditions) are just as or more likely to be motivated to quit than those without. Except for individuals with phobia, smokers with MHCs were less likely to successfully quit, however. The negative association between having any MHC and successful smoking cessation in the previous 12-month period was attenuated after adjustment for heavy smoking. This was not found for individual MHCs; none of which were significantly associated with lower odds of cessation in any the unadjusted or adjusted models. Taken together, despite their higher level of dependence and lower confidence in being able to remain abstinent, and after accounting for number of cigarettes smoked per day, these findings provide evidence against the common misconceptions among healthcare practitioners that people with mental health problems are less motivated and less likely to quit successfully (Sheals, Tombor, McNeill, & Shahab, 2016).

Limitations of the study included the fact that only individuals in private households were interviewed, thus excluding certain groups which typically have a higher prevalence of MHCs and smoking such as those who are homeless or residing in institutions such as prisons or long-term mental health facilities (Jochelson & Majrowski, 2006). Due to data limitations, the study's definition of ex-smokers may have overestimated the size of this group as it may have included individuals who had only smoked just one cigarette previously (ONS, 2017). Our population-level estimates of the proportion of ex-smokers may not be comparable with other studies. The statistical analysis of associations between mental health conditions and smoking and cessation outcomes was limited to data from the 2000 wave. The small number of respondents with particular MHCs (e.g. phobia)^8^ may have limited our ability to detect statistically significant associations with the outcomes tested.

To our knowledge, aside from one study which found that individuals who self-reported having mental health conditions had a higher smoking prevalence, higher cigarette consumption and similar desire to quit compared with those not reporting a condition (Szatkowski & McNeill, 2015), this study is the first to investigate smoking and cessation behaviour by presence of MHCs and personality conditions at the population level within the UK. Strengths of our study, which covered a period of 21 years, included its large sample size, representativeness of the population in Great Britain, and availability of data on specific MHCs assessed using validated and standardised clinical criteria. Although no specific condition had a significantly stronger association with any of the outcome measures employed, the study also addresses an important research gap by investigating the associations between specific MHCs and smoking-related behaviours.

Although smoking prevalence has continued to decline in Britain, prevalence among vulnerable groups such as those with comorbid MHCs remains relatively high. Further progress in reducing smoking and its associated health harms is dependent on addressing specific challenges faced by these groups, such as higher nicotine dependence and perceived difficulty of remaining abstinent from cigarettes, to promote successful cessation and reduce initiation. Although several cost-effective pharmacological and non-pharmacological interventions suitable for individuals with MHCs exist (Anthenelli et al., 2016; Peckham, Brabyn, Cook, Tew, & Gilbody, 2017), access remains an issue (Szatkowski & McNeill, 2013). Further work could address differences in access to treatment, help-seeking and quitting behaviours compared to the general population to inform both population-level policies and the design of tailored cessation interventions. In particular, future studies could consider whether specific nicotine delivery devices, pharmacotherapies or other quit aids may be particularly effective for facilitating cessation among individuals with different mental health conditions (or personality conditions), and whether assumptions of health care professionals (for example, around their desire to quit) may pose additional barriers to providing effective support. Our findings underscore the need to address excess smoking prevalence among people with MHCs, and the continuing importance of interventions such as improving identification of smokers in vulnerable groups within primary and psychiatric settings, strengthening referral systems, increasing availability of pharmacotherapy and counselling, provision of advice for carers, hospital smoking bans and very brief advice to accelerate decline in smoking prevalence and bring about convergence with the general population (Britton, 2015; Lawn & Pols, 2005; NICE, 2013; Royal College of Physicians, 2018; Royal College of Physicians, Royal College of Psychiatrists, 2013). Unless disparities in smoking prevalence between those with and without a MHC are addressed, smoking will continue to contribute to health inequalities between these two groups.

## Contributors

SR designed the study, conducted the statistical analysis and drafted the manuscript. AM and LB revised the manuscript for intellectual content. All authors substantively contributed to and approved the final manuscript.

## Conflict of interest

None.

## Role of funding sources

This study was funded by a Cancer Research UK (CRUK)/BUPA Foundation Cancer Prevention Fellowship (C52999/A19748). The authors are members of the UK Centre for Tobacco and Alcohol Studies, a UK Clinical Research Collaboration Public Health Research: Centre of Excellence. Funding from the Medical Research Council, British Heart Foundation, Cancer Research UK, Economic and Social Research Council and the National Institute for Health Research under the auspices of the UK Clinical Research Collaboration is gratefully acknowledged (MR/K023195/1).

## Appendix A Supplementary data

Supplementary material Image 1

## Footnotes

1 Until 2016, the ONS Annual Population Survey defined ex-smokers as those who responded “yes” to the question “have you ever smoked a cigarette, cigar or pipe?” and “no” to the question “do you smoke cigarettes at all nowadays?” It was determined that this method overestimated the prevalence of ex-smokers as it includes respondents who had perhaps only smoked just one cigarette previously in their life. In response to this, the questions have been changed to “have you ever smoked cigarettes regularly?” and “do you smoke cigarettes at all nowadays?”. This has reduced the proportion of the population considered to be “ex-smokers” and also consequently increased the “never smoked” category (ONS, 2017).

2 Only data from the 2000 wave of APMS were employed in the statistical analysis as information on smoking cessation outcomes was only available in 1993 and 2000 and not in subsequent waves.

3 Respondents were defined as having a desire to quit smoking if they had answered “yes” to the question “would you like to give up smoking altogether?” in the 2000 wave of APMS.

4 Respondents were defined as perceiving remaining abstinent from smoking for one day to be difficult if they had answered “fairly difficult” or “difficult” to the question “how easy or difficult would you find it to go without smoking for a whole day?” in the 2000 wave of APMS.

5 Smoking cessation outcomes were measured among all respondents who were regular smokers at any time within the past 12 months. Respondents were defined as having successfully quit in the last 12 months if they answered “yes” to the questions “have you ever smoked a cigarette?” and “did you smoke cigarettes regularly, that is at least one cigarette a day?”, “no” to the question “do you smoke cigarettes at all nowadays?”, and “6 months but less than a year ago” or “less than 6 months ago” to the question “how long ago did you stop smoking cigarettes regularly?” Respondents who had quit one year ago or more were excluded from the analysis of cessation outcomes.

6 Time to first cigarette after waking was only available for current smokers. Models adjusted for heavy smoking, which was available for both current and ex-smokers, instead of HSI score as the latter was not available for respondents with successful 12-month cessation outcomes.

7 Of the 7,306 respondents in the 2000 wave of APMS with data on smoking status and MHCs, 1,371 (18.8%) had any MHC, 237 (3.2%) had depression, 167 (2.3%) had phobia, 395 (5.4%) had GAD, 64 (0.9%) had panic disorder, 110 (1.5%) had OCD and 692 (9.5%) had mixed anxiety and depression.

8 Of the 7306 respondents in the 2000 wave of APMS with data on smoking status and MHCs, 1371 (18.8%) had any MHC, 237 (3.2%) had depression, 167 (2.3%) had phobia, 395 (5.4%) had GAD, 64 (0.9%) had panic disorder, 110 (1.5%) had OCD and 692 (9.5%) had mixed anxiety and depression.
