# Corrigendum to ‘Smoking and quitting behaviours by mental health conditions in Great Britain (1993–2014)’

**DOI:** 10.1016/j.addbeh.2019.04.005

**Published:** 2019-06

**Authors:** Sol Richardson, Ann McNeill, Leonie S. Brose

**Affiliations:** a King's College London, Addictions Department, Institute of Psychiatry, Psychology & Neuroscience, 4 Windsor Walk, London SE5 8BB, United Kingdom; b UK Centre for Tobacco and Alcohol Studies, United Kingdom

The authors regret to inform readers that a minor error has been identified in the results of this manuscript's descriptive analysis.

This error relates to estimates of weighted proportions of smokers, ex-smokers and never-smokers in the general population and among those with and without a mental health condition (MHC), and the proportion of smokers in each wave with a MHC (16–64 years). The authors wish to inform readers that the 2007 and 2014 waves of the Adult Psychiatric Morbidity Survey (APMS) comprise data from **England only** (data from Scotland and Wales were not available) and not Great Britain (as incorrectly indicated in the manuscript's text).

It should therefore be noted that the proportions originally indicated in the descriptive results for the aforementioned indicators for 2007 and 2014 are correct for England only while results for 1993 and 2000 are correct for Great Britain. For the purpose of their comparability between different years when including data from 2007 and 2014, the correct proportions for **England only** are quoted below for each of the four waves (with proportions from 1993 and 2000 amended to exclude respondents living in Scotland and Wales):

Smoking prevalence in the general population **in England** decreased from 31.6% in 1993 to 22.3% in 2014, and from 29.1% to 19.6% for those without a MHC. Prevalence among those with a MHC **in England** among those with fell from 44.6% to 34.1% in the same period.

The proportion of smokers **in England** who had a MHC increased over the period studied from 22.5% (95% CI: 20.9–24.2) in 1993 to 25.1% (95% CI: 23.1–27.2) in 2000, 25.7% (95% CI: 23.2–28.2) in 2007, and 28.8% (95% CI: 25.9–31.7) in 2014.

The proportion of ex-smokers as a proportion of ever-smokers **in England** increased over time (increasing from 41.8% to 49.4% among those with a MHC and from 58.7% to 66.2% among those without a MHC from 1993 to 2014).

Although these amendments do not change the overall interpretation of the study's descriptive results, nor have any bearing on the validity and accuracy of the results of the statistical analysis (which relate to associations between MHCs and smoking and quitting behaviours for participants **in Great Britain**, and are based on data from the 2000 wave of APMS), the authors would like to apologise for any possible inconvenience caused. Finally, we wish to thank Sally McManus, based at the Health Team at NatCen Social Research, for drawing the authors' attention to this oversight.

