# Supplementary material for: Smoking and quitting behaviours by mental health conditions in Great Britain (1993–2014)
Source: Addict Behav. 2019 Mar;90:14–9. doi: 10.1016/j.addbeh.2018.10.011 (PMC6334164; doi:10.1016/j.addbeh.2018.10.011)
Supplement: Supplementary file 1 — Supplementary material [file mmc1.docx]

# Materials and methods

Respondents were categorised as having a personality condition if they met the criteria for any of the following personality disorders on the Structured Clinical Interview for DSM-IV Personality Disorders (SCID-II) (First et al., 1997; Singleton et al., 2002): avoidant personality disorder, dependent personality disorder, obsessive-compulsive disorder, paranoid personality disorder, schizotypal personality disorder, schizoid personality disorder, histrionic personality disorder, narcissistic personality disorder, borderline personality disorder or antisocial personality disorder.

We investigated associations between having any personality condition and each personality condition with a prevalence of ≥3% in the available analytic sample (avoidant, obsessive-compulsive, paranoid, schizoid and antisocial)^1^ and each smoking-related outcome. Associations were tested using logistic regression and data from the 2000 wave of APMS.

# Results

Having any personality condition was associated with current smoking, heavy smoking, cigarette dependence and perceiving abstinence as difficult (Table 1A). Tables 1A and 1B show the results for the associations between each separate personality condition and smoking-related outcomes. Avoidant personality disorder was associated with higher cigarette dependence and both higher desire to quit smoking and perceived greater difficulty in remaining abstinent among current smokers. Obsessive-compulsive personality disorder was not significantly associated with any of the smoking-related outcomes tested except for successful cessation among respondents who had been smokers within the last 12 months. Having either paranoid or schizoid personality disorder was only significantly associated with higher odds of current smoking or moderate or high cigarette dependence. Antisocial personality disorder was associated with current smoking, and, among smokers, higher odds of perceiving cessation to be difficult.

| Table 1A**. Odds of current smoking, heavy smoking, dependence (moderate or high HSI score), desire to quit and successful smoking cessation by presence of personality conditions among individuals aged 16–64 years in the Adult Psychiatric Morbidity Survey (2000)** | | | | | | |
| --- | --- | --- | --- | --- | --- | --- |
| **i. Current smoking (all respondents, n=6,890)** | | | | | | |
| **Model** | **Any personality disorder^1^** | | **Avoidant** | | **Obsessive-compulsive** | |
|  | **OR (95% CI)** | **p** | **OR (95% CI)** | **p** | **OR (95% CI)** | **p** |
| **Model 1 (unadjusted)** | 1.53 (1.36–1.72) | <0.001 | 1.50 (1.18–1.90) | 0.001 | 0.87 (0.72–1.04) | 0.128 |
| **Model 2^2^** | 1.30 (1.15–1.48) | <0.001 | 1.08 (0.84–1.39) | 0.547 | 1.01 (0.83–1.22) | 0.951 |
| **ii. Heavy smoking (current smokers, n=2,215)** | | | | | | |
| **Model** | **Any personality disorder** | | **Avoidant** | | **Obsessive-compulsive** | |
|  | **OR (95% CI)** | **p** | **OR (95% CI)** | **p** | **OR (95% CI)** | **p** |
| **Model 1 (unadjusted)** | 1.57 (1.28–1.92) | <0.001 | 1.58 (1.07–2.33) | 0.021 | 1.45 (1.04–2.01) | 0.026 |
| **Model 2^2^** | 1.39 (1.12–1.73) | 0.003 | 1.48 (0.97–2.27) | 0.068 | 1.33 (0.95–1.86) | 0.097 |
| **iii. Dependence (moderate or high HSI score) (current smokers, n=2,211)** | | | | | | |
| **Model** | **Any personality disorder** | | **Avoidant** | | **Obsessive-compulsive** | |
|  | **OR (95% CI)** | **p** | **OR (95% CI)** | **p** | **OR (95% CI)** | **p** |
| **Model 1 (unadjusted)** | 1.81 (1.49–2.21) | <0.001 | 2.79 (1.90–4.10) | <0.001 | 1.39 (1.01–1.91) | 0.043 |
| **Model 2^2^** | 1.56 (1.26–1.93) | <0.001 | 2.48 (1.64–3.75) | <0.001 | 1.31 (0.94–1.82) | 0.111 |
| **iv. Desire to quit (current smokers, n=2,202)** | | | | | | |
| **Model** | **Any personality disorder** | | **Avoidant** | | **Obsessive-compulsive** | |
|  | **OR (95% CI)** | **p** | **OR (95% CI)** | **p** | **OR (95% CI)** | **p** |
| **Model 1 (unadjusted)** | 1.00 (0.80–1.24) | 0.967 | 1.91 (1.20–3.05) | 0.007 | 1.12 (0.78–1.61) | 0.527 |
| **Model 2^2^** | 0.96 (0.77–1.20) | 0.731 | 1.79 (1.12–2.87) | 0.015 | 1.10 (0.77–1.58) | 0.594 |
| **Model 3^3^** | 0.96 (0.77–1.20) | 0.708 | 1.78 (1.11–2.86) | 0.016 | 1.10 (0.77–1.58) | 0.605 |
| **v. Difficulty remaining abstinent (n=2,204)** | | | | | | |
| **Model** | **Any personality disorder** | | **Avoidant** | | **Obsessive-compulsive** | |
|  | **OR (95% CI)** | **p** | **OR (95% CI)** | **p** | **OR (95% CI)** | **p** |
| **Model 1 (unadjusted)** | 1.62 (1.33–1.98) | <0.001 | 3.14 (2.00–4.92) | <0.001 | 1.31 (0.95–1.81) | 0.101 |
| **Model 2^2^** | 1.51 (1.22–1.87) | <0.001 | 2.84 (1.75–4.59) | <0.001 | 1.25 (0.89–1.76) | 0.202 |
| **Model 3** | 1.43 (1.14–1.78) | 0.002 | 2.74 (1.68–4.46) | <0.001 | 1.17 (0.82–1.67) | 0.378 |
| **vi. 12-month quit ratio (ever-smokers in last 12 months, n=3,729)** | | | | | | |
| **Model** | **Any personality disorder** | | **Avoidant** | | **Obsessive-compulsive** | |
|  | **OR (95% CI)** | **p** | **OR (95% CI)** | **p** | **OR (95% CI)** | **p** |
| **Model 1 (unadjusted)** | 1.00 (0.75–1.33) | 0.978 | 0.58 (0.29–1.17) | 0.126 | 1.38 (0.91–2.08) | 0.130 |
| **Model 2^2^** | 1.02 (0.75–1.39) | 0.898 | 0.54 (0.26–1.10) | 0.091 | 1.50 (0.98–2.28) | 0.059 |
| **Model 3** | 1.10 (0.81–1.51) | 0.534 | 0.59 (0.29–1.24) | 0.166 | 1.66 (1.08–2.56) | 0.020 |
| ^1^Including depressive episode phobia, generalised anxiety disorder, obsessive compulsive disorder, panic disorder and mixed anxiety and depressive disorder. ^2^Adjusted for gender (binary: male or female), current age (categorical: <25, 25–34, 35–44, 45–54 or 55–64), ethnicity (binary: white or other), partnership status (categorical: married, single, separated, divorced or widowed), housing tenure (categorical: outright ownership, ownership with outstanding mortgage, or rented/other), occupational position (binary: Registrar-General's classification manual or non-manual occupation), level of education (categorical: degree-level, higher vocational (i.e. HND, teaching), A-level, GCSE or equivalent, or no qualifications) and age when first started smoking (categorical: never smoked regularly, <10, 10–14, 15–19, 20–24 or >25). ^3^Adjusted for all covariates included in Model 2 in addition to heavy smoking (binary: <20 or ≥20 cigarettes/weekday). | | | | | | |

| Table 1B**. Odds of current smoking, heavy smoking, dependence (moderate or high HSI score), desire to quit and successful smoking cessation by presence of personality conditions among individuals aged 16–64 years in the Adult Psychiatric Morbidity Survey (2000)** | | | | | | |
| --- | --- | --- | --- | --- | --- | --- |
| **i. Current smoking (all respondents, n=6,890)** | | | | | | |
| **Model** | **Paranoid** | | **Schizoid** | | **Antisocial** | |
|  | **OR (95% CI)** | **p** | **OR (95% CI)** | **p** | **OR (95% CI)** | **p** |
| **Model 1 (unadjusted)** | 1.97 (1.62–2.39) | <0.001 | 1.49 (1.27–1.74) | <0.001 | 3.87 (2.94–5.09) | <0.001 |
| **Model 2^2^** | 1.40 (1.14–1.73) | 0.001 | 1.24 (1.05–1.48) | 0.013 | 2.67 (2.00–3.57) | <0.001 |
| **ii. Heavy smoking (current smokers, n=2,215)** | | | | | | |
| **Model** | **Paranoid** | | **Schizoid** | | **Antisocial** | |
|  | **OR (95% CI)** | **p** | **OR (95% CI)** | **p** | **OR (95% CI)** | **p** |
| **Model 1 (unadjusted)** | 1.18 (0.87–1.60) | 0.280 | 1.64 (1.27–2.12) | <0.001 | 1.39 (0.97–1.98) | 0.073 |
| **Model 2^2^** | 1.17 (0.84–1.63) | 0.343 | 1.30 (0.98–1.71) | 0.065 | 1.10 (0.74–1.63) | 0.637 |
| **iii. Dependence (moderate or high HSI score) (current smokers, n=2,211)** | | | | | | |
| **Model** | **Paranoid** | | **Schizoid** | | **Antisocial** | |
|  | **OR (95% CI)** | **p** | **OR (95% CI)** | **p** | **OR (95% CI)** | **p** |
| **Model 1 (unadjusted)** | 1.82 (1.36–2.44) | <0.001 | 1.87 (1.45–2.40) | <0.001 | 1.33 (0.94–1.89) | 0.108 |
| **Model 2^2^** | 1.78 (1.30–2.44) | <0.001 | 1.38 (1.06–1.80) | 0.016 | 1.02 (0.67–1.53) | 0.943 |
| **iv. Desire to quit (current smokers, n=2,202)** | | | | | | |
| **Model** | **Paranoid** | | **Schizoid** | | **Antisocial** | |
|  | **OR (95% CI)** | **p** | **OR (95% CI)** | **p** | **OR (95% CI)** | **p** |
| **Model 1 (unadjusted)** | 1.17 (0.82–1.65) | 0.386 | 1.23 (0.92–1.64) | 0.161 | 0.88 (0.60–1.30) | 0.528 |
| **Model 2^2^** | 1.18 (0.83–1.67) | 0.368 | 1.18 (0.88–1.60) | 0.271 | 0.83 (0.55–1.25) | 0.379 |
| **Model 3^3^** | 1.17 (0.83–1.67) | 0.372 | 1.18 (0.87–1.60) | 0.281 | 0.83 (0.55–1.25) | 0.376 |
| **v. Difficulty remaining abstinent (n=2,204)** | | | | | | |
| **Model** | **Paranoid** | | **Schizoid** | | **Antisocial** | |
|  | **OR (95% CI)** | **p** | **OR (95% CI)** | **p** | **OR (95% CI)** | **p** |
| **Model 1 (unadjusted)** | 1.43 (1.06–1.95) | 0.021 | 1.37 (1.05–1.77) | 0.018 | 1.62 (1.13–2.34) | 0.010 |
| **Model 2^2^** | 1.39 (1.00–1.92) | 0.050 | 1.08 (0.82–1.42) | 0.588 | 1.57 (1.06–2.33) | 0.024 |
| **Model 3** | 1.37 (0.97–1.93) | 0.075 | 0.99 (0.74–1.33) | 0.962 | 1.60 (1.06–2.42) | 0.025 |
| **vi. 12-month quit ratio (ever-smokers in last 12 months, n=3,729)** | | | | | | |
| **Model** | **Paranoid** | | **Schizoid** | | **Antisocial** | |
|  | **OR (95% CI)** | **p** | **OR (95% CI)** | **p** | **OR (95% CI)** | **p** |
| **Model 1 (unadjusted)** | 1.18 (0.75–1.85) | 0.475 | 0.77 (0.51–1.16) | 0.209 | 0.75 (0.41–1.39) | 0.368 |
| **Model 2^2^** | 1.08 (0.69–1.70) | 0.739 | 0.88 (0.58–1.35) | 0.561 | 0.62 (0.33–1.16) | 0.134 |
| **Model 3** | 1.18 (0.74–1.88) | 0.486 | 0.90 (0.58–1.38) | 0.619 | 0.72 (0.37–1.40) | 0.327 |
| ^1^Including depressive episode phobia, generalised anxiety disorder, obsessive compulsive disorder, panic disorder and mixed anxiety and depressive disorder. ^2^Adjusted for gender (binary: male or female), current age (categorical: <25, 25–34, 35–44, 45–54 or 55–64), ethnicity (binary: white or other), partnership status (categorical: married, single, separated, divorced or widowed), housing tenure (categorical: outright ownership, ownership with outstanding mortgage, or rented/other), occupational position (binary: Registrar-General's classification manual or non-manual occupation), level of education (categorical: degree-level, higher vocational (i.e. HND, teaching), A-level, GCSE or equivalent, or no qualifications) and age when first started smoking (categorical: never smoked regularly, <10, 10–14, 15–19, 20–24 or >25). ^3^Adjusted for all covariates included in Model 2 in addition to heavy smoking (binary: <20 or ≥20 cigarettes/weekday). | | | | | | |

# References

First, M. B., Gibbon, M., Spitzer, R. L., William, J. B. W., & Benjamin, L. (1997). Structured Clinical Interview for DSM–IV Axis II Personality Disorders. Washington: American Psychiatric Press.

Singleton, N., Bumpstead, R., O’Brien, M., Lee, A., Meltzer, H. (2002). Psychiatric morbidity among adults living in private households 2000. London: Her Majesty's Stationery Office (HMSO).

# Footnotes

1. Of the 7,162 respondents in the 2000 wave of APMS with data on smoking status and personality conditions, 383 (5.4%) had avoidant personality disorder, 96 (1.3%) had dependent personality disorder, 739 (10.3%) had obsessive-compulsive disorder, 573 (8.0%) had paranoid personality disorder, 213 (3.0%) had schizotypal personality disorder, 940 (13.1%) had schizoid personality disorder, 48 (0.7%) had histrionic personality disorder, 31 (0.4%) had narcissistic personality disorder, 109 (1.5%) had borderline personality disorder, and 286 (4.0%) had antisocial personality disorder.
